# Supplementary material for: Resilience Against COVID-19: How Italy Faced the Pandemic in Pediatric Orthopedics and Traumatology
Source: Children (Basel). 2021 Jun 22;8(7):530. doi: 10.3390/children8070530 (PMC8305147; doi:10.3390/children8070530)
Supplement: Supplementary file 1 [file children-08-00530-s001.zip › children-1242707-supplementary.pdf]

Table S1. Activity comparison between 2019 vs 2018 period.

	**Year**		2019 vs 2018 comparison*		
Year	2018	2019	Percent relative change	95%CI	p
Total Surgery	8283	8628	4.2%	1.1% to 7.4%	0.008
High-Priority	3970	4221	6.3%	1.8% to 11%	0.006
Trauma Surgery	2394	2608	8.9%	3.1% to 15.2%	0.002
Priority A	1257	1295	3%	-4.7% to 11.3%	0.452
Priority B	319	318	-0.3%	-14.7% to 16.4%	0.968
Low-Priority	4313	4407	2.2%	-2% to 6.6%	0.314
Priority C	3577	3653	2.1%	-2.5% to 6.9%	0.371
Priority D	736	754	2.4%	-7.4% to 13.4%	0.641
Outpatient Visits	68569	71837	4.8%	3.7% to 5.9%	<0.001
Emergency visits	42954	42649	-0.7%	-2% to 0.6%	0.297

Table S2. 2020 vs 2018-19 Activity comparisons, by single hospital.

**Hospital***	**Procedure**	**2018**	**2019**	**2020**	**Percent relative change 2020 vs 2018-19**	**95%CI**	**p**
Hospital 1 (S,H,E)	Trauma surgery	788	828	700	-13.4%	-20.7% to -5.3%	0.002
	Elective surgery	437	548	569	15.5%	4.2% to 28.1%	0.006
	Outpatient Visits	17949	18746	15319	-16.5%	-18.1% to -14.9%	<0.001
	ED Visits	4236	4054	4574	10.3%	6.4% to 14.4%	<0.001
Hospital 2 (N,L,T)	Trauma surgery	228	218	191	-14.3%	-27.7% to 1.5%	0.073
	Elective surgery	786	880	632	-24.1%	-30.8% to -16.9%	<0.001
	Outpatient Visits	13923	14358	10404	-26.4%	-28.1% to -24.8%	<0.001
	ED Visits	9880	9833	5262	-46.6%	-48.2% to -45%	<0.001
Hospital 3 (S,L,E)	Trauma surgery	196	216	154	-25.2%	-37.9% to -10%	0.002
	Elective surgery	314	355	285	-14.8%	-25.8% to -2.1%	0.024
	Outpatient Visits	9610	8760	7046	-23.3%	-25.4% to -21.2%	<0.001
	ED Visits	3205	3376	2563	-22.1%	-25.6% to -18.5%	<0.001
Hospital 4 (N,L,T)	Trauma surgery	134	172	159	3.9%	-14.2% to 25.9%	0.694
	Elective surgery	581	508	286	-47.5%	-53.9% to -40.2%	<0.001
	Outpatient Visits	3000	3000	1600	-46.7%	-49.5% to -43.6%	<0.001
	ED Visits	4542	4585	2420	-47%	-49.3% to -44.5%	<0.001
Hospital 5 (N,H,T)	Trauma surgery	167	194	141	-21.9%	-35.7% to -5.1%	0.013
	Elective surgery	3116	3137	1967	-37.1%	-40.2% to -33.8%	<0.001
	Outpatient Visits	13405	14560	12301	-12%	-13.9% to -10.1%	<0.001
	ED Visits	7701	7861	4206	-45.9%	-47.8% to -44.1%	<0.001
Hospital 6 (S,H,E)	Trauma surgery	881	980	815	-12.4%	-19.3% to -4.9%	0.002
	Elective surgery	470	386	412	-3.7%	-14.4% to 8.3%	0.525
	Outpatient Visits	9498	11232	7532	-27.3%	-29.2% to -25.4%	0
	ED Visits	13390	12940	7046	-46.5%	-47.9% to -45.1%	0
Hospital 7 (N,L,T)	Trauma surgery	0	0	0	-	-	-
	Elective surgery	185	206	142	-27.4%	-40.1% to -12%	0.001
	Outpatient Visits	1184	1181	984	-16.8%	-22.7% to -10.4%	<0.001
	ED Visits	0	0	0	-	-	-

* Each hospital was classified according to the following categories: S = Southern; N = Northern; H = High Volume; L = Low Volume; T = mainly trauma surgery; E = mainly elective surgery.

Table S3. 2020 vs 2018-19 Activity comparisons by geographic location.

**Outcome**	**Geographic location**	**2018**	**2019**	**2020**
Surgery	Southern	3086	3313	2935
	Northern	5197	5315	3518
Outpatient visits	Southern	37057	38738	29897
	Northern	31512	33099	25289
ED visits	Southern	20831	20370	14183
	Northern	22123	22279	11888

Table S4. 2020 vs 2018-19 Activity comparisons by center characteristics.

Outcome	Center Characteristic	Relative change	95%CI	p-interaction	LR test Chi-2*
Surgery	Southern	-8.3%	-12.2% to -4.2%	<0.001	7.16
	Northern	-33.1%	-35.6% to -30.5%		
	Low-volume surgery	-25.7%	-29.6% to -21.7%	0.236	112.17
	High-volume surgery	-22.8%	-25.4% to -20.2%		
	Elective predominant	-33.1%	-35.6% to -30.5%	<0.001	6.95
	Trauma predominant	-8.3%	-12.2% to -4.2%		

* Likelihood-ratio test comparing mixed effect Poisson model with random slope for center vs random intercept only. LR test chi-2= 112.73 for the model that does not include any center characteristics.

Table S5. Percentage relative change "high-priority" vs "low-priority" comparing 2020 vs 2018-2019 period.

Quarter	Outcome	Relative change	95%CI	p-interaction*
All quarters	High-priority	-17.2%	-20.4% to -13.8%	<0.001
	Low-priority	-29.8%	-32.6% to -26.8%	
1 MAR-31 MAY	High-priority	-39.5%	-44.8% to -33.8%	<0.001
	Low-priority	-60.3%	-64.2% to -56%	
1 JUN-31 AUG	High-priority	-1.1%	-7.8% to 6%	<0.001
	Low-priority	-29.6%	-35.4% to -23.4%	
1 SEP-30 NOV	High-priority	-4.1%	-11.1% to 3.5%	0.005
	Low-priority	-17.6%	-23.6% to -11.1%	
1 DIC-28 FEB	High-priority	-28.8%	-35.1% to -22%	0.001
	Low-priority	-12.6%	-18.9% to -5.8%	

*Mixed effects Poisson regression model.

Table S6. Percentage relative change elective surgery vs. outpatient visits comparing 2020 vs 2018-2019 period.

Quarter	Outcome	Relative change	95%CI	p-interaction*
All quarters	Elective Surgery	-27.9%	-30.4% to -25.3%	<0.001
	Outpatient visits	-21.4%	-22.2% to -20.6%	
1MAR-31MAY	Elective Surgery	-56.8%	-60.4% to -53%	0.973
	Outpatient visits	-56.8%	-57.8% to -55.7%	
1JUN-31AUG	Elective Surgery	-22.5%	-27.7% to -16.8%	<0.001
	Outpatient visits	-7.1%	-8.9% to -5.3%	
1SEP-30NOV	Elective Surgery	-14.3%	-19.7% to -8.6%	0.098
	Outpatient visits	-9.4%	-11% to -7.7%	
1DIC-28FEB	Elective Surgery	-18.1%	-23.3% to -12.6%	0.002
	Outpatient visits	-9%	-10.7% to -7.2%	

* Mixed effects Poisson regression model.

Table S7. Percentage relative change emergency surgery vs ED visit comparing 2020 vs 2018-2019 period.

Quarter	Outcome	Relative change	95%CI	p-interaction*
All quarters	Emergency Surgery	-13.6%	-17.9% to -9.2%	<0.001
	ED Visits	-39.1%	-39.9% to -38.2%	
1MAR-31MAY	Emergency Surgery	-34.1%	-41.2% to -26.2%	<0.001
	ED Visits	-65.2%	-66.3% to -64.1%	
1JUN-31AUG	Emergency Surgery	-0.2%	-8.2% to 8.6%	<0.001
	ED Visits	-15%	-17.1% to -12.8%	
1SEP-30NOV	Emergency Surgery	-3.6%	-12.4% to 6.1%	<0.001
	ED Visits	-30.3%	-32.1% to -28.5%	
1DIC-28FEB	Emergency Surgery	-25%	-33.8% to -15%	<0.001
	ED Visits	-40.6%	-42.4% to -38.8%	

*Mixed effects Poisson regression model.
